# Integrating Functional Analysis in the Next-Generation Sequencing Diagnostic Pipeline of RASopathies

**DOI:** 10.1038/s41598-018-20894-0

**Published:** 2018-02-05

**Authors:** Gordon K. C. Leung, H. M. Luk, Vincent H. M. Tang, W. W. Gao, Christopher C. Y. Mak, Mullin H. C. Yu, W. L. Wong, Yoyo W. Y. Chu, W. L. Yang, Wilfred H. S. Wong, Alvin C. H. Ma, Anskar Y. H. Leung, D. Y. Jin, Kelvin Y. K. Chan, Judith Allanson, Ivan F. M. Lo, Brian H. Y. Chung

**Affiliations:** 1 Department of Paediatrics and Adolescent Medicine, Li Ka Shing Faculty of Medicine, The University of Hong Kong, Hong Kong, China; 2 Clinical Genetic Service, Department of Health, Hong Kong, China; 3 School of Biomedical Sciences, Li Ka Shing Faculty of Medicine, The University of Hong Kong, Hong Kong, China; 4 Department of Medicine, Li Ka Shing Faculty of Medicine, The University of Hong Kong, Hong Kong, China; 5 Department of Health Technology and Informatics, The Hong Kong Polytechnic University, Hong Kong, China; 6 Department of Obstetrics and Gynaecology, Tsan Yuk Hospital, Hong Kong, China; 7 Department of Paediatrics, Faculty of Medicine, University of Ottawa, Ontario, Canada

## Abstract

RASopathies are a group of heterogeneous conditions caused by germline mutations in RAS/MAPK signalling pathway genes. With next-generation sequencing (NGS), sequencing capacity is no longer a limitation to molecular diagnosis. Instead, the rising number of variants of unknown significance (VUSs) poses challenges to clinical interpretation and genetic counselling. We investigated the potential of an integrated pipeline combining NGS and the functional assessment of variants for the diagnosis of RASopathies. We included 63 Chinese patients with RASopathies that had previously tested negative for *PTPN11* and *HRAS* mutations. In these patients, we performed a genetic analysis of genes associated with RASopathies using a multigene NGS panel and Sanger sequencing. For the VUSs, we evaluated evidence from genetic, bioinformatic and functional data. Twenty disease-causing mutations were identified in the 63 patients, providing a primary diagnostic yield of 31.7%. Four VUSs were identified in five patients. The functional assessment supported the pathogenicity of the *RAF1* and *RIT1* VUSs, while the significance of two VUSs in *A2ML1* remained unclear. In summary, functional analysis improved the diagnostic yield from 31.7% to 36.5%. Although technically demanding and time-consuming, a functional genetic diagnostic analysis can ease the clinical translation of these findings to aid bedside interpretation.

## Introduction

The application of next-generation sequencing (NGS) has rapidly expanded our knowledge of the mutational and phenotypic spectrum of genetic disorders. The massive parallel sequencing of millions of DNA fragments allows research and clinical laboratories to analyse multiple genes in the entire genome rapidly, and as a result, sequencing capacity is no longer a limitation. With the rapid decline in cost, various types of NGS, including multigene sequencing panels, whole-exome-sequencing (WES), or whole-genome sequencing (WGS), are now in routine clinical use to diagnose genetic disorders^1–3^. In general, gene panels are favoured for genetically heterogeneous conditions in which a clear clinical hypothesis is established before testing^4–6^. Studies have shown that the clinical sensitivity of disease-focused gene panels can actually be higher than WES/WGS^7,8^. In addition, gene panels usually have lower costs, and the data size is more manageable. Therefore, in some clinical laboratories, NGS gene panels are preferred over WES/WGS.

Despite the advantages of gene panels, the interpretation of genotype-phenotype relationships and the consequence of genetic variants are still major challenges to its clinical application. Although significant progress developing bioinformatic prediction tools and databases for international data sharing has been achieved, the interpretation of variants of unknown significance (VUSs) still impedes realizing the full application of genomic medicine. False pathogenicity assignments can have adverse consequences for patients, as genetic diagnosis is now used to manage patient care at different stages of life^2^. Extensive efforts have been made to elucidate the causality of VUSs in human diseases^9–11^. In summary, a two-step process is advised. First, evidence implicating a specific gene should be considered (gene level assessment). Second, a combined assessment of the genetic, computational and experimental support for an individual candidate genetic variant should then be performed (variant level assessment). Clinicians and clinical laboratories are usually more involved in variant assessment, and the usual strategy includes clinical correlation, family segregation, allelic frequency, and computational prediction, as well as some limited functional studies available in clinical settings, including biochemical analysis for metabolic diseases or mRNA studies. More recently, the ClinGen Clinical Validity Framework made a recommendation on experimental evidence, giving more weight to *in vivo* (model systems) compared to *in vitro* (function and functional alteration) experimental data^12^.

RASopathies are a group of developmental conditions caused by germline mutations in genes involved in the RAS/MAPK signalling pathway^13,14^. These conditions are clinically heterogeneous and include Noonan or Noonan-like syndromes, Costello syndrome, cardio-facio-cutaneous syndrome, neurofibromatosis type I and related disorders. They have both unique and overlapping clinical features recognizable by clinical geneticists who usually formulate a diagnostic hypothesis prior to genetic testing^15^. However, they are also genetically heterogeneous illnesses caused by gain-of-function mutations in multiple genes of the pathway, which regulate cell growth, proliferation and differentiation via a signalling cascade involving small GTPases and the phosphorylation of downstream molecules, such as MAPK3 (Legacy symbol: ERK1) and ELK1^16^. Various functional assays have been used for gene discovery, including *in vitro* luciferase assays and *in vivo* zebrafish (*Danio rerio*) modelling^17–20^. The aim of this study was to examine the feasibility of combining an NGS gene panel and functional assays for variant level assessment with an integrated pipeline for the diagnosis of RASopathies.

## Results

### Mutation analysis

**Figure 1:**
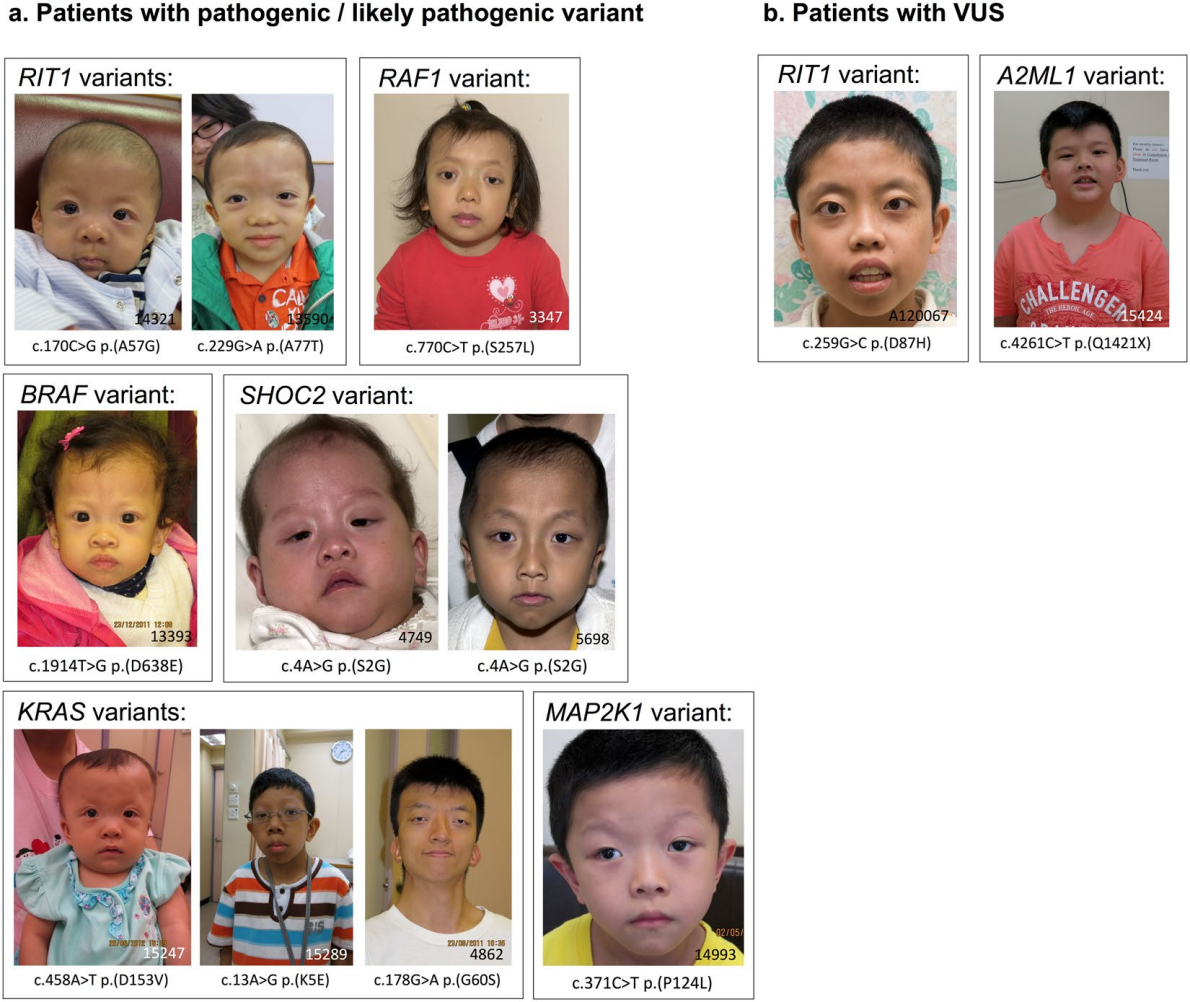
Clinical photographs of patients with variants in genes of the RAS/MAPK signalling pathway. Patients with (**a**) pathogenic or likely pathogenic variants and (**b**) VUSs from our study.

In this study, 20 pathogenic or likely pathogenic mutations were identified in the 63 patients recruited. Of these, 18 were identified in the NGS panel and two were from *RIT1* sequencing, and all were missense mutations. Six (9.5%) were found in *SOS1*; two (3.1%), in *RAF1*; three (4.7%), in *KRAS*; three (4.7%), in *MAP2K1*; two (3.1%), in *RIT1*; two (3.1%), in *BRAF*; and two (3.1%), in *SHOC2*. These mutations have been previously reported in medical literature and various mutation databases (Table 1), and the clinical features of these patients were compatible with RASopathies (Fig. 1 and suppl. Table 1). Combining the NGS panel and *RIT1* sequencing, a molecular diagnosis was established in 31.7% (20/63) of the patients.

**Table 1:** A summary of 20 patients identified with pathogenic or likely pathogenic mutations from our cohort using the multigene panel sequencing approach. *Clinical significance was labelled according to the ClinVar database from NCBI (https://www.ncbi.nlm.nih.gov/clinvar/). ^#^Variant class was determined according to the HGMD (Human Gene Mutation Database http://www.hgmd.cf.ac.uk/docs/new_help.html). ^^^Variant classification was determined according to the NSEuroNet (European Network on Noonan Syndrome and Related Disorders; https://nseuronet.com/php/about.php).

Patient number	Gene	Mutation	ClinVar^*^	HGMD^#^	NSEuroNet^^^	Publication with functional analysis
5132	SOS1	c.512 T > C:p.(V171A)	likely pathogenic	—	—	
15547	SOS1	c.1297 G > A:p.(E433K)	pathogenic	disease-causing mutation	Mutation (count = 19)	
14626	SOS1	c.1644T > G:p.(S548R)	pathogenic	disease-causing mutation	Mutation (count = 1)	Smith *et al*. (2013)
5608	SOS1	c.1644T > A:p.(S548R)	pathogenic	—	—	
6381	SOS1	c.1644T > A:p.(S548R)	pathogenic	—	—	
9233	SOS1	c.1654A > G:p.(R552G)	pathogenic	disease-causing mutation	Mutation (count = 52)	
4748	RAF1	c.770 C > T:p.(S257L)	likely pathogenic	disease-causing mutation	Mutation (count = 73)	
3347	RAF1	c.770 C > T:p.(S257L)	likely pathogenic	disease-causing mutation	Mutation (count = 73)	
15289	KRAS	c.13 A > G:p.(K5E)	likely pathogenic	disease-causing mutation	Mutation (count = 3)	
4862	KRAS	c.178 G > A:p.(G60S)	pathogenic	disease-causing mutation	Mutation (count = 1)	
15247	KRAS	c.458 A > T:p.(D153V)	pathogenic	disease-causing mutation	Mutation (count = 13)	
6575	MAP2K1	c.199 G > A:p.(D67N)	likely pathogenic	—	Mutation (count = 6)	
14993	MAP2K1	c.371 C > T:p.(P124L)	pathogenic	disease-causing mutation	Mutation (count = 1)	Emery *et al*. (2009)
4012	MAP2K1	c.389 A > G:p.(Y130C)	pathogenic	disease-causing mutation	Mutation (count = 35)	Cheng *et al*. (2012)
14321	RIT1	c.170 C > G:p.(A57G)	pathogenic	disease-causing mutation	Mutation (count = 23)	Chen *et al*. (2014)
13590	RIT1	c.229 G > A:p.(A77T)	likely pathogenic	disease-causing mutation	Mutation (count = 6)	
5153	BRAF	c.1785T > G:p.(F595L)	likely pathogenic	disease-causing mutation	Mutation (count = 5)	Cheng *et al*. (2012)
13393	BRAF	c.1914T > G:p.(D638E)	pathogenic	disease-causing mutation	Mutation (count = 5)	Cheng *et al*. (2012)
4749	SHOC2	c.4 A > G:p.(S2G)	likely pathogenic	disease-causing mutation	Mutation (count = 169)	
5698	SHOC2	c.4 A > G:p.(S2G)	likely pathogenic	disease-causing mutation	Mutation (count = 169)

Four VUSs were identified in five patients (7.9%) according to the ACMG guideline^10^. Three were missense mutations, and one was a nonsense mutation. *RAF1*:c.1173 G > C:p.(R391S) was identified in a mother and son, both of whom exhibited features of Noonan syndrome. In addition, one *RIT1* (c.259 G > C:p.(D87H)) and two *A2ML1* (c.256 C > T:p.(P86S), c.4261 C > T:p.(Q1421X)) variants were observed in three other patients. From the bioinformatics analysis, an evaluation of these variants showed stronger evidence of pathogenicity for the *RIT1* and *RAF1* variants than the two *A2ML1* variants (Table 2). The clinical features of the patients carrying these variants are shown in Fig. 1 and in suppl. Table 2.

**Table 2:** A summary of five patients identified with VUSs from our cohort using the multigene panel sequencing approach. *Clinical significance was labelled according to the ClinVar database from NCBI (https://www.ncbi.nlm.nih.gov/clinvar/). ^#^Variant class was determined according to the HGMD (Human Gene Mutation Database http://www.hgmd.cf.ac.uk/docs/new_help.html). ^^^Variant classification was determined according to the NSEuroNet (European Network on Noonan Syndrome and Related Disorders; https://nseuronet.com/php/about.php). ^%^The maximum credible population allelic frequency for Noonan syndrome (1.0 × 10^−4^) was calculated based on disease prevalence, maximum allelic contribution, maximum genetic contribution and penetrance^37^. Allelic frequency data was extracted from ExAC (Exome Aggregation Consortium; http://exac.broadinstitute.org).

Patient number	Gene	Mutation	Clinvar^*^	HGMD^#^	Nseuronet^^^	Allelic frequency in gnomAD	Allelic frequency in ExAC	exceeded maximum credible population allele frequency^%^	GERP score	DANN score	Mutation Taster	PROVEAN	SIFT
4868	*RAF1*	c.1173 G > C:p.(R391S)	—	—	—	Not reported	Not reported	—	4.82	0.9977	Disease-causing	Damaging	Damaging
4869	*RAF1*	c.1173 G > C:p.(R391S)	—	—	—	Not reported	Not reported	—	4.82	0.9977	Disease-causing	Damaging	Damaging
A120067	*RIT1*	c.259 G > C:p.(D87H)	uncertain significance	—	—	Not reported	Not reported	—	5.76	0.9949	Disease-causing	Damaging	Damaging
10235	*A2ML1*	c.256 C > T:p.(P86S)	—	—	—	15 in 245,782 alleles 6.103 × 10^−5^	9 in 119,026 alleles 7.561 × 10^−5^	No	3.99	0.9984	Polymorphism	Damaging	Tolerated
15424	*A2ML1*	c.4261 C > T:p.(Q1421X)	—	—	—	58 in 246,126 alleles 2.357 × 10^−4^	27 in 120,712 alleles 2.237 × 10^−4^	Yes	3.32	0.9952	Disease-causing (automatic)	—	—

### Functional characterization of the VUSs

**Figure 2:**
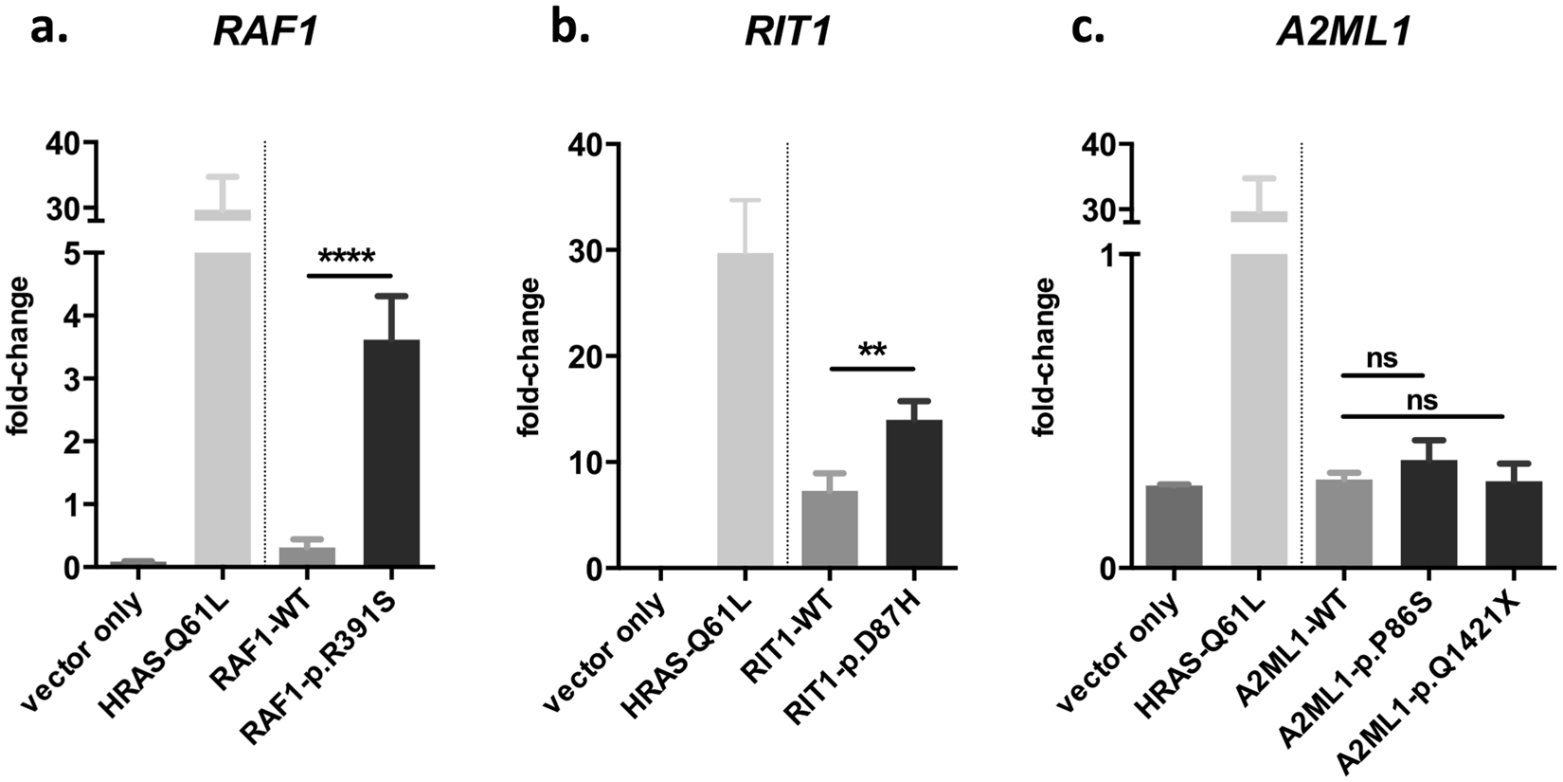
Dual luciferase assay of the phosphorylation activity changes to ELK1 from 293 T cells transfected with the corresponding expression and reporter plasmids. Statistical significance was derived using an unpaired t-test comparing the mutants with the corresponding WT. **p < 0.01; ***p < 0.001; ****p < 0.0001; ns: not significant. Mutated human transcript with VUSs from (**a**) *RAF1* (**b**) *RIT1* and (**c**) *A2ML1* from this study were compared with the wild-type transcript. The data are the mean ± SD from 3 determinations.

To assess the pathogenicity of the four VUSs found in this study, we introduced mutations in the human coding transcript. Previous studies suggest that a majority of mutations causing RASopathies lead to the activation of the RAS/MAPK signalling pathway^17,21,22^. Increased signalling is measured by the level of downstream substrate phosphorylation, such as phosphorylated ELK1 (pELK1). To evaluate whether the RAS/MAPK pathway was activated by the VUSs in our study, we introduced mutant reporter plasmids into HEK-293T cells to assess phosphorylation levels using a dual luciferase reporter assay. Both *RAF1*:p.R391S and *RIT1*:p.D87H significantly increased pELK1 expression compared with the WT transcripts (Fig. 2). In contrast, the two *A2ML1* mutations did not produce a significant fold change in pELK1 levels *in vitro*.

**Figure 3:**
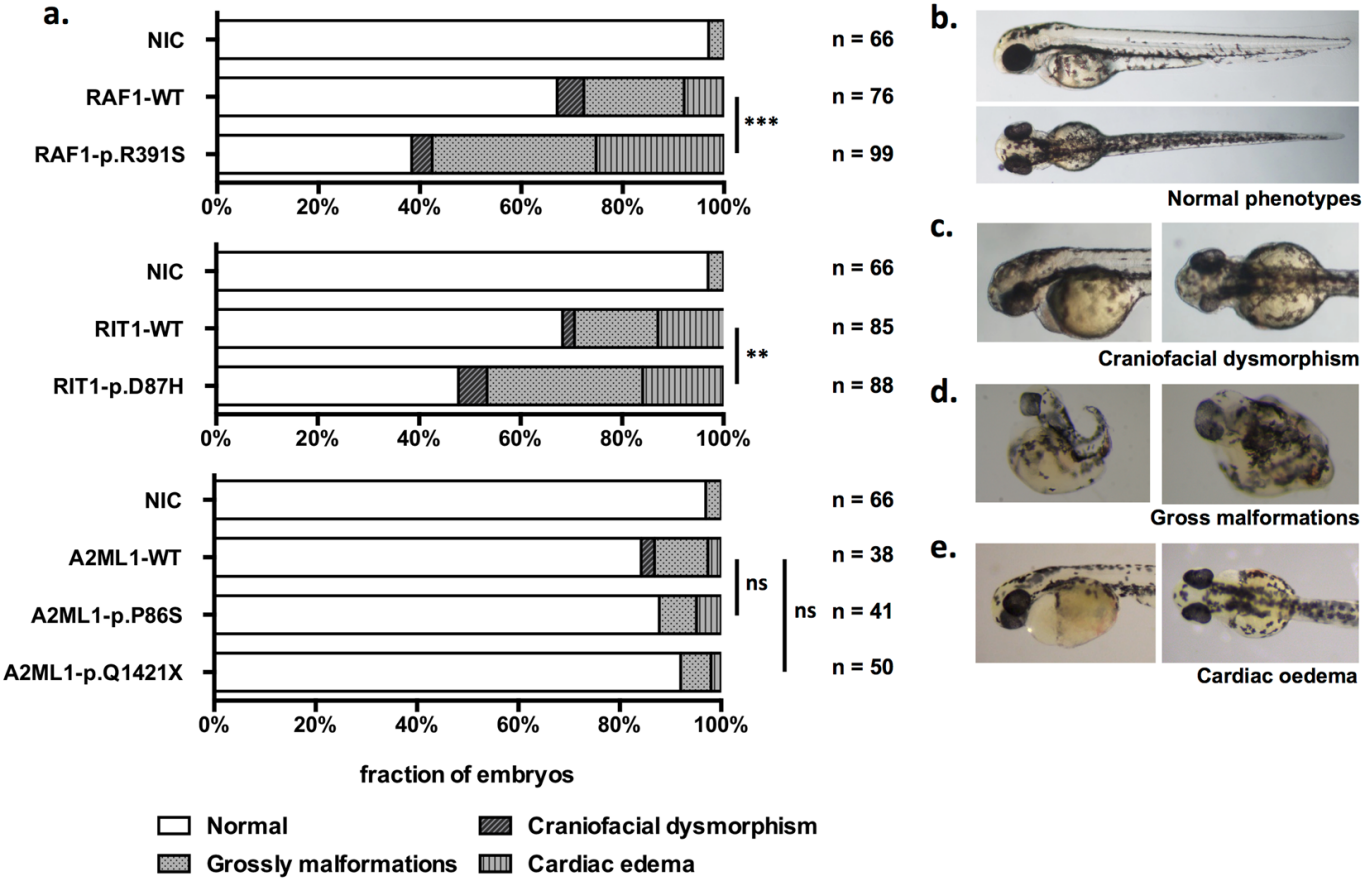
Transient expression of RNA transcripts in zebrafish embryos. The injection dosage of each RNA transcript was optimised for comparison (*RAF1*: 50 pg/embryo; *RIT1*: 400 pg/embryo; *A2ML1*: 200 pg/embryo). A morphometric analysis was performed to compare the effect of the VUSs compared to the wild-type transcript at three dpf. Statistical significance was derived using a two-sided Fisher’s exact test to compare mutants with the corresponding WT. **p < 0.01; ***p < 0.001; ns: not significant. NIC: No injection control; (**a**) Proportion of zebrafish embryos with normal or diseased phenotypes. (**b**) Representative zebrafish embryos with a normal phenotype (**c**) craniofacial dysmorphism (**d**) gross malformations and (**e**) cardiac oedema.

**Figure 4:**
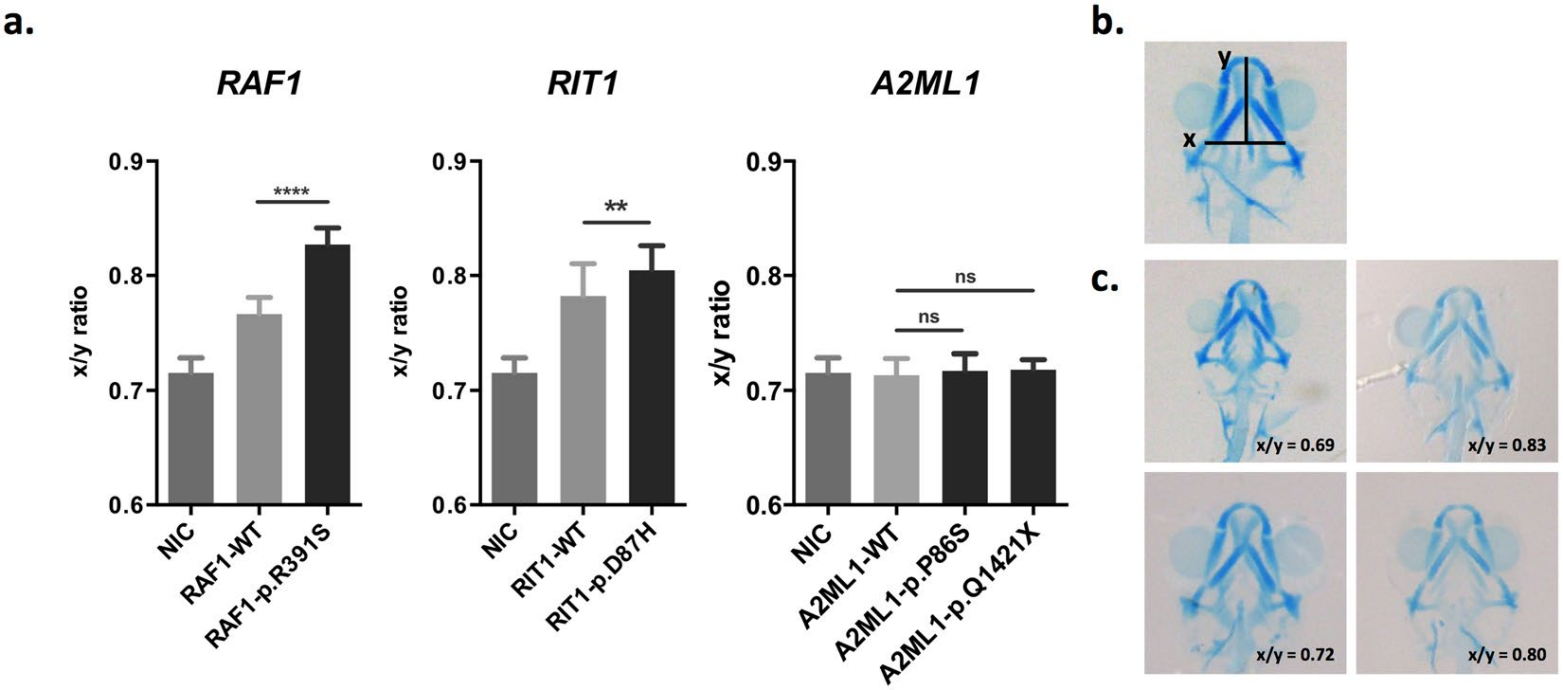
Craniofacial assessment of zebrafish embryos. The zebrafish embryos were treated with Alcian blue and washed with acidic alcohol 3 dpf. The ratio of the width of the ceratohyal (x) to the tip of Meckel’s cartilage (y) was used as a measure of craniofacial defects. (**a**) Comparison of the x-to-y ratio between mutant and wild-type zebrafish embryos. The data are the mean ± SD. Statistical significance was derived using an unpaired t-test comparing the mutants with the corresponding WT. **p < 0.01; ****p < 0.0001; ns: not significant. NIC: No injection control. The number in bold represents the total count of zebrafish embryos in the experimental group. (**b**) Alcian-blue stained cartilage in the zebrafish. The x-axis shows the width of the ceratohyal, and the y-axis shows the length of the tip of Meckel’s cartilage. (**c**) Representative embryos with different x-to-y ratios.

**Figure 5:**
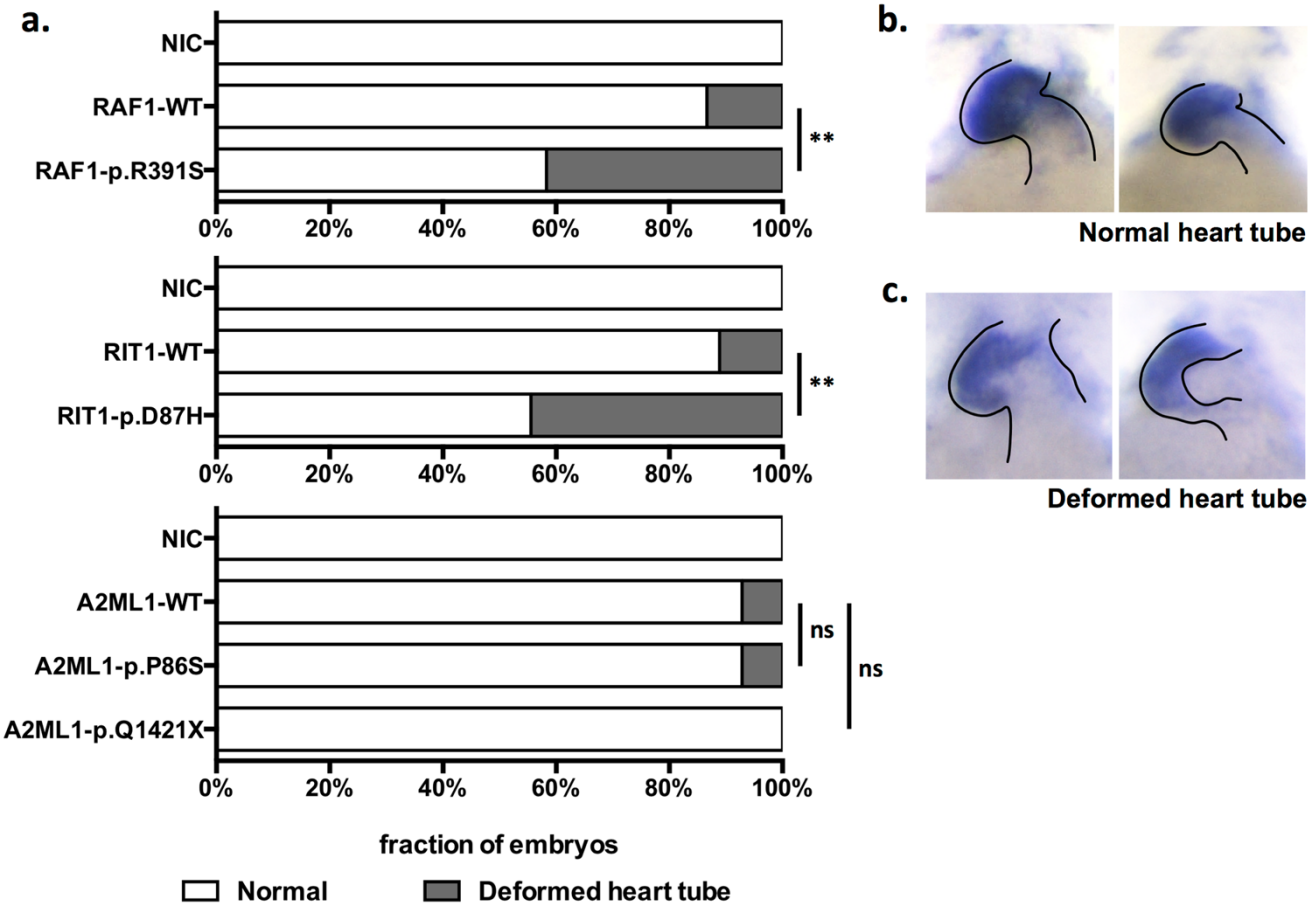
Structural assessment of cardiac tubes by *in situ* hybridization of *cmlc1*. Statistical significance was derived using a two-sided Fisher’s exact test to compare mutants with the corresponding WT. **p < 0.01; ns: not significant. (**a**) The proportion of zebrafish embryos with a normal or deformed heart tube structure. (**b**) Representative embryos with a normal and (**c**) deformed heart tube.

The phenotypic impact of the VUSs on embryonic development was examined by transiently expressing WT or mutant transcripts in zebrafish embryos at the single-cell stage. We scored the embryos according to their morphological appearance (Fig. 3), the width-to-length ratio of the head (Fig. 4), and *in situ* hybridization of the cardiac marker *cmlc1* (Fig. 5). These features correspond to the typical phenotypes of Noonan syndrome (cranio-facial dysmorphism and cardiac abnormalities) in human patients. The morphometric analysis agreed with the results from the luciferase assay, and the activation of the RAS signals in the zebrafish embryos was also compatible with the phenotypes (data not shown). A significant portion of zebrafish embryos injected with human mRNA transcripts with novel mutations in *RAF1* and *RIT1* developed atypical craniofacial and heart structures, whereas the expression of the *A2ML1* mutations did not affect zebrafish development.

In summary, both *in vitro* and *in vivo* analyses provided further supportive evidence for the functional significance of the *RAF1* and *RIT1* variants, whereas the significance of the two VUSs in *A2ML1* remained unclear. A combined assessment of the genetic, bioinformatic and experimental evidence from the *RAF1* and *RIT1* variants led to a re-classification of the VUSs as likely pathogenic mutations, which increased the overall diagnostic yield from 31.7% to 36.5%.

## Discussion

This study aimed to establish a diagnostic pipeline for RASopathies that integrates an NGS panel with functional genetics. We adopted the experimental approach used for gene discovery applied to *RAF1*, *RIT1* and *A2ML1*^17–19^ on variant assessment for VUSs in these genes such that the same criteria can be used to judge their pathogenicity. Our initial diagnostic yield was 31.7%, which was lower than that of other RASopathy studies (diagnostic yield of 60–70%)^7,15^, because the patients recruited had already tested negative for *PTPN11* and *HRAS* mutations. The functional genetic studies contributed to resolving two of four VUSs, increasing the diagnostic yield from 31.7% to 36.5%.

For the four VUSs we identified, *in silico* predictions suggested that the variants were either disease-causing or damaging in nature (Table 2). For the *RAF1*:p.R391S variant, it was located at an evolutionarily conserved genomic locus at conserved region 3 (CR3) of *RAF1* gene. CR3 encodes multiple protein kinase catalytic domains for ATP binding^23,24^; however, p.R391S does not directly interfere with the functional motif of CR3. The phenotype-genotype correlation between Noonan syndrome and the *RAF1* mutation were previously reported^19,21^, and the two patients in our cohort with the p.R391S mutation had typical features of Noonan syndrome. *RIT1*:p.D87H was reported once previously as a VUS in the ClinVar database. Our patient with the *RIT1* mutation presented with typical facial features of Noonan syndrome (Fig. 1), as well as hypertrophic cardiomyopathy (HCM) and sub-aortic stenosis. Additional features included congenital chylothorax, multifocal bronchomalacia, and bilateral lower-limb lymphangiectasia of the calves. The variant p.D87H is not located in one of the five functional domains of *RIT1*. Still, pathogenic mutations for Noonan syndrome have been reported in an amino acid position adjacent to p.D87H^17^. In patients with *RIT1* mutations, HCM (56%), atrial defects, and pulmonary stenosis are the more frequently reported clinical manifestations^25^. The clinical presentation of our patient was consistent with these published phenotypes and included cardiac anomalies and lymphatic complications^26,27^. Therefore, combining all of the evidence and functional analysis, we classified the two genetic variants *RAF1*:p.R391S and *RIT1*:p.D87H as likely pathogenic mutations, according to the latest guidelines^9,10,12^.

The genetic variants *A2ML1*:p.P86S and *A2ML1*:p.Q1421X did not appear functionally significant after *in vitro* or *in vivo* assessment. To date, the association of *A2ML1* with Noonan-related syndrome has only been reported in one study^18^. Vissers *et al*. identified a *de novo* variant of *A2ML1* in an individual with Noonan syndrome by WES, and additional analyses of 155 individuals revealed two more families with Noonan syndrome carrying *A2ML1* variants. From the current understanding, the majority of reported pathogenic mutations in RASopathies are gain-of-function mutations, whereas one of the two *A2ML1* variants in our study is a null mutation. Constraint analysis from ExAC suggested that heterozygous loss-of-function variants in *A2ML1* are common across the gene in unaffected populations^28^, and intragenic deletions have also been reported in normal individuals^29^. Therefore, it is unlikely that *A2ML1* mutations act via haploinsufficiency. An independent assay, similar to the methodology used in the first publication on *A2ML1*^18^ was used for functional analysis. We illustrated that the two *A2ML1* variants in our cohort did not activate the RAS/MAPK pathway, and they did not affect the development of the zebrafish embryos. Our findings suggested that the functional significance of the two *A2ML1* variants might not affect the RAS/MAPK pathway via gain-of-function nor dominant negative mechanism, and the clinical significance of the two variants remains unclear. This observation is actually in line with Aoki *et al*.^30^, stating that the functional properties of *A2ML1* and the mechanisms by which *A2ML1* regulates the RAS/MAPK pathway is largely unknown. Furthermore, the phenotypes of the two patients were reviewed by a highly experienced clinical geneticist who is an internationally recognized expert in RASopathies, blinded to the functional analysis and computational prediction. It was concluded that both patients in our cohort did not have the classical phenotype of Noonan syndrome (clinical photograph of one patient is shown in Fig. 1).

Herein, we used a multigene sequencing panel to identify the disease-causing mutations in patients with RASopathies with a primary diagnostic yield of 31.7%. We also performed a functional characterisation of VUSs and confirmed the pathogenic nature of two. The inclusion of functional data improved the diagnostic yield by 4.8%. As suggested by ACMG guidelines, well-established functional studies can serve as a strong evidence of pathogenicity (i.e. PS3). Integrating robust functional analysis into routine clinical laboratory tests is challenging yet feasible. Some of these challenges include the requirement of specialised expertise and collaboration with research laboratories (e.g., zebrafish facilities). The knowledge of disease-causing mechanisms is also essential and experiments can only be designed for known disease pathways or known structural changes (e.g. ciliopathies). It may seem practically difficult to apply this one-by-one approach to the large number of genetic variants discovered by WES and WGS. However, a recent report showed that a multiplexed functional assay can be used to map sequence-function relationships with base-pair resolution for hundreds to thousands of variants in both coding and non-coding regions of the genome in a single experiment^31^. This innovation can potentially overcome the current limitations of functional genetics and can be implemented successfully into the clinical diagnostic pipeline in the near future.

Molecular diagnosis using NGS can facilitate the clinical management for patients with genetic diseases. The concept of predictive genomics has been reviewed in clinical oncology, in which the genomic data of tumor was used to predict clinical phenotypes and tumor progression to better design patient treatment^32^. Based on a systematic approach, we could predict personalized drug targets and outcome for cancer patients^33^. For patients with other genetic syndromes, timely diagnosis and predictable clinical outcomes are always beneficial to the patient^34^. Therefore, the establishment of functional analysis for genetic syndromes would help clinical decision-making in the long term and facilitate comprehensive interpretation of new variants by clinicians.

## Materials and Methods

### Patient recruitment

This study was approved by the Institutional Review Board of the University of Hong Kong/ Hospital Authority Hong Kong West Cluster (HKU/HA HKW IRB). Sixty-three Chinese patients presenting with clinical suspicion of RASopathies were recruited from the Clinical Genetics Service, Department of Health, Hong Kong, and Department of Paediatrics and Adolescent Medicine, the University of Hong Kong, Hong Kong. These subjects were evaluated by clinical geneticists from the two centres. Because *PTPN11* and *HRAS* sequencing were available at these clinics, all 63 subjects were tested and were negative for mutations in these two genes. Informed consent was obtained from the parents and/or patients for the participation, and publication of identifying images in this study.

### Next generation sequencing

Genomic DNA was extracted from patient peripheral blood using standard protocols. We designed a gene enrichment panel for 13 genes known to cause RASopathies in 2013, which included *A2ML1* (NM_144670.4)*, BRAF* (NM_004333.4)*, CBL* (NM_005188.3)*, HRAS* (NM_005343.3)*, KRAS* (NM_004985.3)*, MAP2K1* (NM_002755.3)*, MAP2K2* (NM_030662.3)*, NRAS* (NM_002524.4)*, PTPN11* (NM_002834.3)*, RAF1* (NM_002880.3)*, SHOC2* (NM_007373.3), *SOS1* (NM_005633.3), and *SPRED1* (NM_152594.2). The panel covered the coding exons (flanked by ±25 bp) of these 13 selected genes (suppl. Figure 1). The size of the final target region was 101.38 kbp with 97.99% coverage of the target region. Target sequencing was performed using the Haloplex custom target enrichment system (Agilent Technologies, CA, USA) on the MiSeq sequencing platform with the MiSeq reagent kit V2. The average read depth in the regions of interest was >500×, and over 98% of the covered region had a sequencing depth of at least 30× . The data were analysed using NextGENe software v.2.3.4.1 (Softgenetics, PA, USA) with the default settings. Regions with low coverage (<20× read depth) were re-sequenced by Sanger sequencing. Genetic variant pathogenicity was assessed according to the guidelines for the interpretation of sequence variants suggested by the American College of Medical Genetics and Genomics (ACMG)^10^. Disease-causing mutations were Sanger validated. During the study period, *RIT1* (NM_006912.4) was identified as a new causative gene for Noonan syndrome^17^. We subsequently designed primers and sequenced the five coding exons of *RIT1*.

### Functional analysis

Genetic variants classified as VUSs were selected for functional characterization. The VUSs were *RAF1*:NM_002880.2:c.1173 G > C:p.(R391S), *A2ML1*:NM_144670.4:c.256 C > T:p.(P86S), *A2ML1*:NM_144670.4:c.4261 C > T:p.(Q1421X) and *RIT1*:NM_006912.4:c.259 G > C:p.(D87H). Expression plasmids for *RAF1* (#RC201983), *A2ML1* (#RC219615) and *RIT1* (#RC220552) were ordered from Origene (MD, USA). We introduced a single-base substitution into the corresponding expression vector using Q5® Site-Directed Mutagenesis Kit (NEB, UK). The *RAF1*, *A2ML1* and *RIT1* coding sequences were validated using Sanger sequencing before subsequent analysis.

### *In vitro* dual luciferase reporter assay

To evaluate the molecular effect of the VUSs identified in the cohort, mutant transcripts were expressed in HEK-293T cells. The phosphorylation level of ELK1, which is a downstream substrate of the RAS/MAPK pathway, was measured as a reporter of pathway activity^17,35^. HEK-293T cells were cultured in DMEM and transfected with expression plasmids using Genejuice transfection reagent (EMD Chemicals, NJ, USA). We introduced pGal-LUC (Clontech), pGal-ELK1 and the expression plasmids into HEK-293T cells. The expression plasmids included the mutant constructs of *RAF1*, *A2ML1* and *RIT1*, or the corresponding wild-type gene vectors for comparison. In each experimental setup, an empty vector was used as the negative control, while a HRAS expression plasmid with p.Q61L mutation was used as a positive control^36^. The cells were harvested 48 hours post-transfection. The luciferase activity was measured using a Dual-Luciferase® Reporter assay system (Promega, WI, USA). Transfection efficiencies were normalised to the control plasmid, which was pSV-RLuc (Promega, WI, USA) expressing *Renilla* luciferase. The expression of the DDK-tagged transcript was validated by Western blot using anti-DDK antibodies.

### *In vivo* transient expression in zebrafish embryos

To assess the effect of the VUSs on organismal phenotypes, zebrafish were used according to the methods previously described in experiments assessing gene level evidence^9–11^. Briefly, the in-frame coding sequence of *RAF1*, *A2ML1* and *RIT1* were subcloned into the pCMV6-AC-GFP vector such that the GFP coding sequence was fused in-frame to the 3′ end of the inserted gene. GFP-tagged RNA transcripts were synthesised using the mMESSAGE mMACHINE T7 Transcription Kit (Thermo Fisher Scientific, MA, USA). RNA transcripts were injected into zebrafish embryos at the single-cell stage. GFP signals in the zebrafish embryos indicated synthetic RNA expression and served as a positive selection marker for morphometric assessment. Phenotypic observation was performed 3 days post-fertilization (dpf), as previously described^18^. The embryos were anesthetised with tricane methanesulfonate and fixed in 4% paraformaldehyde (PFA), and their general body structure and the development of craniofacial and cardiac structure were assessed. Cartilage was stained with 0.1% Alcian Blue in acidic ethanol. The width and length of the cranio-cartilage were measured using ImageJ software. The ratio of the width of the ceratohyal to the tip of Meckel’s cartilage was determined as a measure of craniofacial defects. For cardiac defects, PFA fixed embryos were probed with digoxigenin-labelled *cmlc1*, which is a cardiac development marker for zebrafish, by *in situ* hybridization. Total protein from zebrafish embryos was also extracted to examine the correlation between the zebrafish phenotypes and the activation of RAS signals. The study was approved by the Committee of the Use of Live Animals for Teaching and Research in The University of Hong Kong.

### Data availability

The datasets generated during and/or analysed during the current study are available from the corresponding author on reasonable request.

## Electronic supplementary material

Supplementary figure 1

Supplementary table 1a

Supplementary table 1b
