# Supplementary material for: Integrating Functional Analysis in the Next-Generation Sequencing Diagnostic Pipeline of RASopathies
Source: Sci Rep. 2018 Feb 5;8:2421. doi: 10.1038/s41598-018-20894-0 (PMC5799236; doi:10.1038/s41598-018-20894-0)
Supplement: Supplementary file 1 — Supplementary figure 1 [file 41598_2018_20894_MOESM1_ESM.docx]

**Integrating Functional Analysis in the Next-Generation Sequencing Diagnostic Pipeline of RASopathies – Supplementary Information**

Gordon KC Leung^1^, HM Luk^2^, Vincent HM Tang^3^, WW Gao^3^, Christopher CY Mak^1^, Mullin HC Yu^1^, WL Wong^1^, Yoyo WY Chu^1^, WL Yang^1^, Wilfred HS Wong^1^, Alvin CH Ma^4,5^, Anskar YH Leung^4^, DY Jin^3^, Kelvin YK Chan^6^, Judith Allanson^7^, Ivan FM Lo^2^* and Brian HY Chung^1,6^*

Affiliations:

^1^ Department of Paediatrics and Adolescent Medicine, Li Ka Shing Faculty of Medicine, The University of Hong Kong, Hong Kong, China

^2^ Clinical Genetic Service, Department of Health, Hong Kong, China

^3^ School of Biomedical Sciences, Li Ka Shing Faculty of Medicine, The University of Hong Kong, Hong Kong, China

^4^ Department of Medicine, Li Ka Shing Faculty of Medicine, The University of Hong Kong, Hong Kong, China

^5^ Department of Health Technology and Informatics, The Hong Kong Polytechnic University, Hong Kong, China

^6^ Department of Obstetrics and Gynaecology, Tsan Yuk Hospital, Hong Kong, China

^7^ Department of Paediatrics, Faculty of Medicine, University of Ottawa, Ontario, Canada

* Co-correspondence:

Dr. Lo Ivan Fai-Man, Clinical Genetic Service, Department of Health, Hong Kong, China. 3/F, Cheung Sha Wan Jockey Club Clinic, 2 Kwong Lee Road, Sham Shui Po, Kowloon, Hong Kong. Tel.: (+852) 2304-2063. Fax: (+852) 2729-1440. Email: [dr.ivanlo@gmail.com](mailto:dr.ivanlo@gmail.com)

Dr. Chung Brian Hon-Yin, Department of Paediatrics and Adolescent Medicine, Li Ka Shing Faculty of Medicine, The University of Hong Kong, Hong Kong, China. Rm 103, 1/F, New Clinical Building, Queen Mary Hospital, Hong Kong. Tel.: (+852) 2255-4482. Fax: (+852) 2855-1523. Email: [bhychung@hku.hk](mailto:bhychung@hku.hk)

**Supplementary figure 1: The target gene panel design of our enrichment panel.**

**
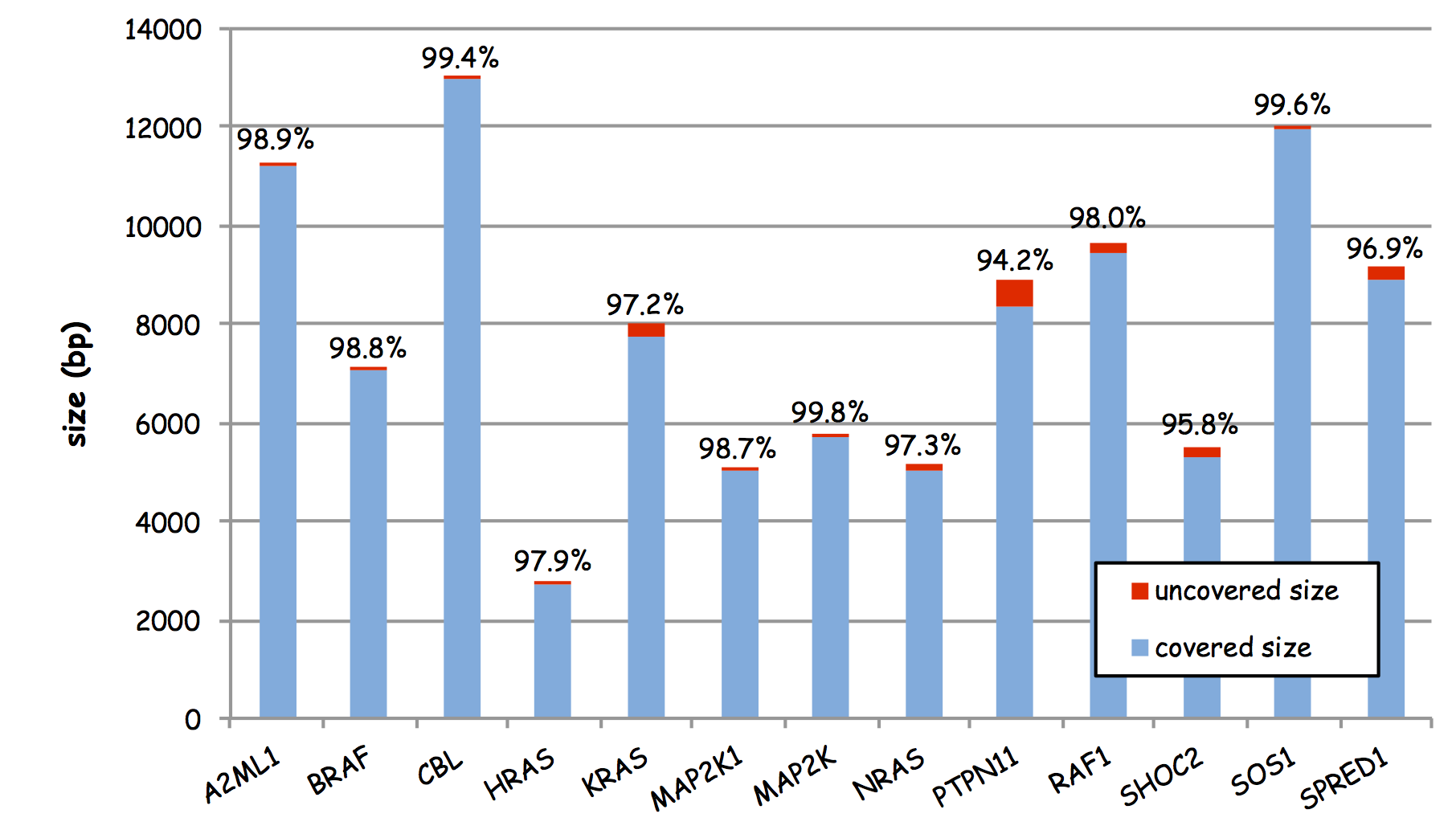
**
